# Diisopropyl [(4-meth­oxy­benzamido)(*p*-tol­yl)meth­yl]phospho­nate

**DOI:** 10.1107/S1600536814004413

**Published:** 2014-03-12

**Authors:** Hua Fang, Guang-Qin Wang, Wei-Zhu Chen, Rui-Zao Yi, Zhuan Hong

**Affiliations:** a The Third Institute of Oceanography of the State Oceanic Administration, Xiamen 361005, People’s Republic of China

## Abstract

The asymmetric unit of the title compound, C_22_H_30_NO_5_P, contains two independent mol­ecules in which the dihedral angles between the benzene rings are 82.0 (2) and 78.4 (2)°. In the crystal, each mol­ecule forms an inversion dimer *via* a pair of N—H⋯O(=P) hydrogen bonds.

## Related literature   

For a related structure, see: Giarda *et al.* (1973 ▶).
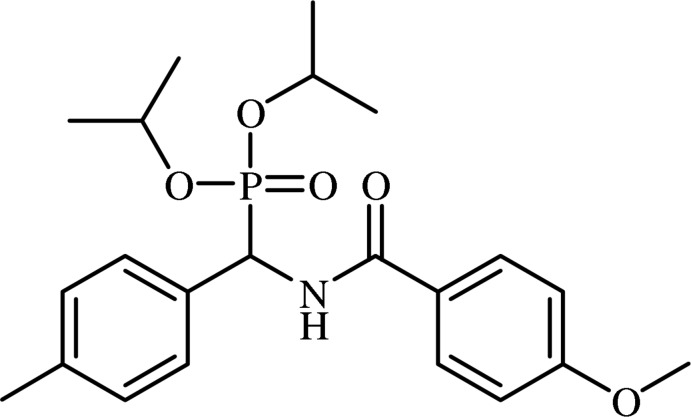


## Experimental   

### 

#### Crystal data  

C_22_H_30_NO_5_P
*M* *_r_* = 419.44
Triclinic, 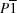

*a* = 12.4900 (7) Å
*b* = 14.0338 (8) Å
*c* = 14.5693 (9) Å
α = 73.389 (5)°
β = 76.324 (5)°
γ = 78.609 (5)°
*V* = 2354.5 (2) Å^3^
*Z* = 4
Mo *K*α radiation
μ = 0.15 mm^−1^
*T* = 293 K
0.44 × 0.32 × 0.18 mm

#### Data collection  

Bruker APEX CCD diffractometer
Absorption correction: multi-scan (*SADABS*; Bruker, 2001 ▶) *T*_min_ = 0.938, *T*_max_ = 0.974
17787 measured reflections
7970 independent reflections
4850 reflections with *I* > 2σ(*I*)
*R*_int_ = 0.048

#### Refinement  

*R*[*F* ^2^ > 2σ(*F* ^2^)] = 0.067
*wR*(*F* ^2^) = 0.237
*S* = 1.08
7970 reflections
523 parameters
H-atom parameters constrained
Δρ_max_ = 0.37 e Å^−3^
Δρ_min_ = −0.24 e Å^−3^

### 

Data collection: *SMART* (Bruker, 2001 ▶); cell refinement: *SAINT* (Bruker, 2001 ▶); data reduction: *SAINT*; program(s) used to solve structure: *SHELXS97* (Sheldrick, 2008 ▶); program(s) used to refine structure: *SHELXL97* (Sheldrick, 2008 ▶); molecular graphics: *PLATON* (Spek, 2009 ▶); software used to prepare material for publication: *SHELXL97*.

## Supplementary Material

Crystal structure: contains datablock(s) I. DOI: 10.1107/S1600536814004413/lh5691sup1.cif

Structure factors: contains datablock(s) I. DOI: 10.1107/S1600536814004413/lh5691Isup2.hkl

Supporting information file. DOI: 10.1107/S1600536814004413/lh5691Isup3.cml

CCDC reference: 988872

Additional supporting information:  crystallographic information; 3D view; checkCIF report

## Figures and Tables

**Table 1:** Hydrogen-bond geometry (Å, °)

*D*—H⋯*A*	*D*—H	H⋯*A*	*D*⋯*A*	*D*—H⋯*A*
N1—H1*A*⋯O2^i^	0.86	2.20	3.021 (4)	159
N2—H2*A*⋯O7^ii^	0.86	2.20	3.013 (4)	157

Symmetry codes: (i) 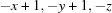; (ii) 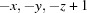.

**Fig. 1.**
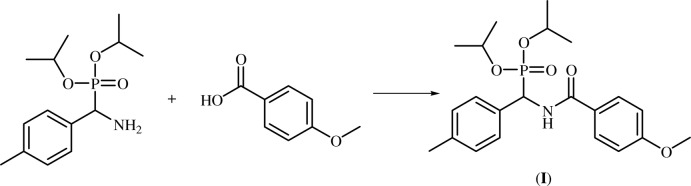
The reaction scheme for the formation of the title compound.

**Fig. 2.**
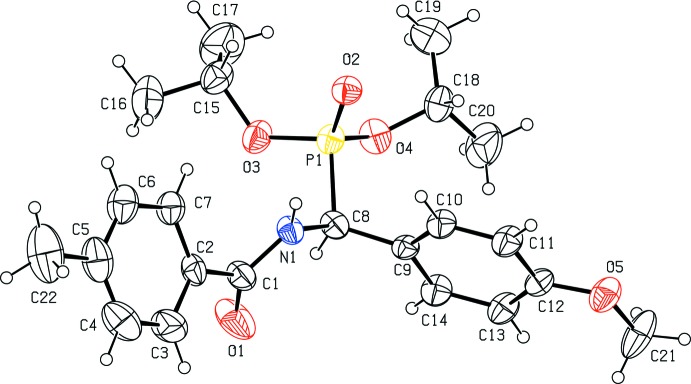
The molecular structure of one independent molecule. Displacement ellipsoids are drawn at the 30% probability level and H atoms are shown as spheres of arbitrary radii.

**Fig. 3.**
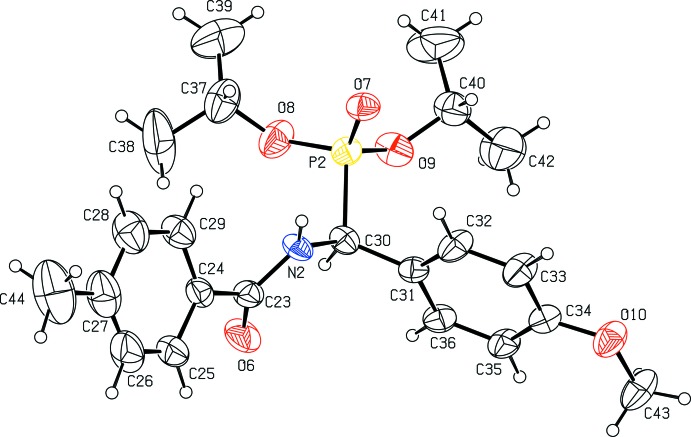
The molecular structure of the other independent molecule. Displacement ellipsoids are drawn at the 30% probability level and H atoms are shown as spheres of arbitrary radii.

**Fig. 4.**
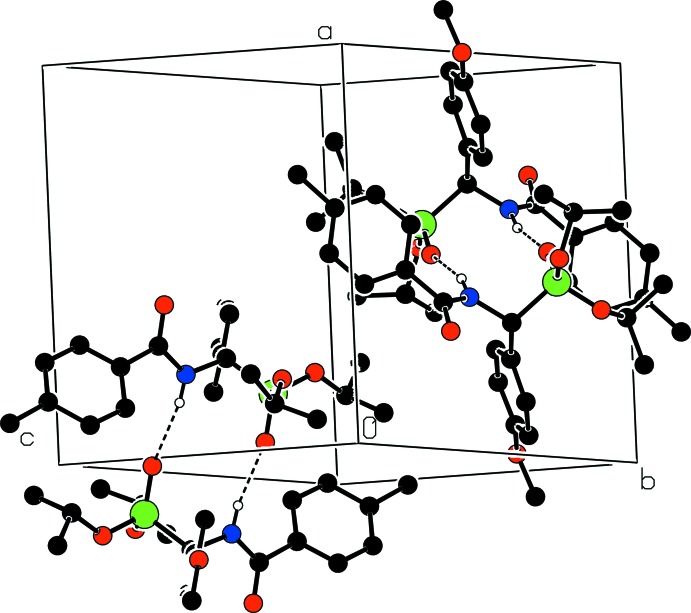
Part of the crystal structure with hydrogen bonds shown as dashed lines.

## supplementary crystallographic information

### 1. Comment

The title compound, (I), was synthesized for a study of its antimicrobial activity against Bacillus subtilis. The hydroxyphosphonic acid derivative was found to have moderate antimicrobial activity (inhibition zone = 10 mm). The adjacent bonds P1—O3 and P1—O4 [mean 1.557 (8) Å] are similar to that in ammonium dimethylphosphate [1.559 (7) Å; Giarda *et al.*, 1973]. The reaction scheme for (I) is shown in Fig. 1.

The title compound (I) contains two independent molecules which are shown in Figs. 2 and 3. The dihedral angle between the benzene rings in each are 82.0 (2) ° [C2-C7/C9-C14] and 78.4 (2) ° [C23-C28/C30-C35]. In the crystal, each molecule forms an inversion dimer via a pair of N—H···O(═P) hydrogen bonds (Fig. 4).

### 2. Experimental

Triphenylphosphine (0.39 g, 1.5 mmol) and hexachloroethane (0.39 g, 1.2 mmol) in dried 1,2-dichloroethane (15 ml) were reacted for 1 h in an ice bath. The resulting solution was added dropwise to a mixture of the hydrochloride of dialkyl α-aminotolylphosphonate (0.28 g, 1.0 mmol) and 4-methoxybenzoic acid (0.15 g, 1.0 mmol) in 1,2-dichloroethane (10 ml) and triethylamine (0.4 ml). After completion of the reaction, the solvent was removed to give the crude product, which was purified by recrystallization from hot ethanol. Single crystals of (I) suitable for X-ray analysis were obtained by slow evaporation of a petroleum ether C ethyl/acetate solution (1:1 v/v) of the title compound. Analysis calculated for C_22_H_30_NO_5_P: C 63.00, H 7.21, N 3.34; Found C 63.38, H 7.16, N 3.39.

### 3. Refinement

The H atoms were positioned geometrically (C—H = 0.93, 0.98 or 0.96 A for benzene, tertiary or methyl H atoms, respectively, and N—H = 0.86 Å) and were included in the refinement in the riding-model approximation. The displacement parameters of the methyl H atoms were set at 1.5 *U*_eq_ of their parent atoms, while those of the other H atoms were set at 1.2 *U*_eq_(C,N).

### Crystal data

**Table:**

C_22_H_30_NO_5_P	*Z* = 4
*M**_r_* = 419.44	*F*(000) = 896
Triclinic, *P*1	*D*_x_ = 1.183 Mg m^−^^3^
Hall symbol: -P 1	Mo *K*α radiation, λ = 0.71073 Å
*a* = 12.4900 (7) Å	Cell parameters from 1986 reflections
*b* = 14.0338 (8) Å	θ = 1.3–27.1°
*c* = 14.5693 (9) Å	µ = 0.15 mm^−^^1^
α = 73.389 (5)°	*T* = 293 K
β = 76.324 (5)°	Block, colorless
γ = 78.609 (5)°	0.44 × 0.32 × 0.18 mm
*V* = 2354.5 (2) Å^3^	

### Data collection

**Table:**

Bruker APEX CCD diffractometer	7970 independent reflections
Radiation source: fine-focus sealed tube	4850 reflections with *I* > 2σ(*I*)
Graphite monochromator	*R*_int_ = 0.048
φ and ω scans	θ_max_ = 25.0°, θ_min_ = 3.1°
Absorption correction: multi-scan (*SADABS*; Bruker, 2001)	*h* = −14→14
*T*_min_ = 0.938, *T*_max_ = 0.974	*k* = −16→16
17787 measured reflections	*l* = −16→17

### Refinement

**Table:**

Refinement on *F*^2^	Primary atom site location: structure-invariant direct methods
Least-squares matrix: full	Secondary atom site location: difference Fourier map
*R*[*F*^2^ > 2σ(*F*^2^)] = 0.067	Hydrogen site location: inferred from neighbouring sites
*wR*(*F*^2^) = 0.237	H-atom parameters constrained
*S* = 1.08	*w* = 1/[σ^2^(*F*_o_^2^) + (0.1456*P*)^2^] where *P* = (*F*_o_^2^ + 2*F*_c_^2^)/3
7970 reflections	(Δ/σ)_max_ < 0.001
523 parameters	Δρ_max_ = 0.37 e Å^−^^3^
0 restraints	Δρ_min_ = −0.24 e Å^−^^3^

### Special details

**Table:**

Geometry. All e.s.d.'s (except the e.s.d. in the dihedral angle between two l.s. planes) are estimated using the full covariance matrix. The cell e.s.d.'s are taken into account individually in the estimation of e.s.d.'s in distances, angles and torsion angles; correlations between e.s.d.'s in cell parameters are only used when they are defined by crystal symmetry. An approximate (isotropic) treatment of cell e.s.d.'s is used for estimating e.s.d.'s involving l.s. planes.
Refinement. Refinement of *F*^2^ against ALL reflections. The weighted *R*-factor *wR* and goodness of fit *S* are based on *F*^2^, conventional *R*-factors *R* are based on *F*, with *F* set to zero for negative *F*^2^. The threshold expression of *F*^2^ > σ(*F*^2^) is used only for calculating *R*-factors(gt) *etc*. and is not relevant to the choice of reflections for refinement. *R*-factors based on *F*^2^ are statistically about twice as large as those based on *F*, and *R*- factors based on ALL data will be even larger.

### Fractional atomic coordinates and isotropic or equivalent isotropic displacement parameters (Å^2^)

**Table:**

	*x*	*y*	*z*	*U*_iso_*/*U*_eq_	
P1	0.57811 (7)	0.44284 (7)	0.16751 (6)	0.0520 (3)	
N1	0.6169 (2)	0.6091 (2)	0.0288 (2)	0.0516 (7)	
H1A	0.5685	0.6027	−0.0015	0.062*	
C1	0.6337 (3)	0.7016 (3)	0.0261 (3)	0.0638 (10)	
O1	0.7087 (3)	0.7155 (2)	0.0598 (3)	0.1190 (14)	
O2	0.49931 (17)	0.41867 (18)	0.11966 (17)	0.0571 (6)	
C2	0.5546 (3)	0.7864 (2)	−0.0178 (3)	0.0582 (9)	
P2	0.14534 (7)	0.06980 (7)	0.34174 (7)	0.0566 (3)	
N2	0.1934 (2)	−0.0972 (2)	0.4752 (2)	0.0547 (7)	
H2A	0.1239	−0.0969	0.5007	0.066*	
O3	0.52694 (19)	0.5103 (2)	0.2420 (2)	0.0708 (7)	
C3	0.5913 (4)	0.8747 (3)	−0.0739 (4)	0.0982 (16)	
H3A	0.6668	0.8799	−0.0872	0.118*	
O4	0.6468 (2)	0.35073 (19)	0.22714 (18)	0.0659 (7)	
C4	0.5166 (6)	0.9569 (4)	−0.1114 (5)	0.1155 (19)	
H4A	0.5432	1.0156	−0.1505	0.139*	
O5	0.98353 (19)	0.2768 (2)	−0.1426 (2)	0.0767 (8)	
C5	0.4038 (5)	0.9519 (4)	−0.0909 (4)	0.0962 (15)	
C6	0.3690 (4)	0.8631 (4)	−0.0355 (4)	0.0823 (12)	
H6A	0.2934	0.8580	−0.0219	0.099*	
O6	0.3615 (2)	−0.1907 (2)	0.4513 (3)	0.0923 (10)	
C7	0.4419 (3)	0.7802 (3)	0.0013 (3)	0.0644 (10)	
H7A	0.4151	0.7208	0.0385	0.077*	
O7	0.02795 (18)	0.08346 (19)	0.38750 (19)	0.0636 (7)	
C8	0.6792 (3)	0.5189 (2)	0.0822 (3)	0.0517 (8)	
H8A	0.7198	0.5406	0.1208	0.062*	
O8	0.1754 (2)	0.0138 (3)	0.2580 (2)	0.0911 (10)	
C9	0.7636 (2)	0.4594 (2)	0.0191 (2)	0.0462 (7)	
O9	0.2008 (2)	0.1666 (2)	0.2895 (2)	0.0786 (8)	
C10	0.7348 (3)	0.4320 (3)	−0.0546 (3)	0.0550 (9)	
H10A	0.6639	0.4541	−0.0690	0.066*	
O10	0.2849 (2)	0.2332 (3)	0.6678 (2)	0.0842 (9)	
C11	0.8103 (3)	0.3722 (3)	−0.1069 (3)	0.0651 (10)	
H11A	0.7896	0.3544	−0.1564	0.078*	
C12	0.9168 (2)	0.3378 (3)	−0.0877 (3)	0.0550 (9)	
C13	0.9486 (3)	0.3677 (3)	−0.0166 (3)	0.0632 (10)	
H13A	1.0201	0.3474	−0.0035	0.076*	
C14	0.8705 (3)	0.4291 (3)	0.0351 (3)	0.0618 (9)	
H14A	0.8918	0.4503	0.0823	0.074*	
C15	0.4143 (3)	0.5126 (4)	0.2970 (3)	0.0760 (12)	
H15A	0.3680	0.4918	0.2625	0.091*	
C16	0.3747 (5)	0.6195 (5)	0.2997 (5)	0.1170 (19)	
H16A	0.3785	0.6609	0.2343	0.176*	
H16B	0.4209	0.6407	0.3320	0.176*	
H16C	0.2992	0.6255	0.3345	0.176*	
C17	0.4119 (6)	0.4438 (6)	0.3946 (5)	0.140 (2)	
H17A	0.4374	0.3763	0.3885	0.210*	
H17B	0.3371	0.4478	0.4314	0.210*	
H17C	0.4595	0.4622	0.4277	0.210*	
C18	0.6608 (3)	0.2496 (3)	0.2166 (3)	0.0709 (11)	
H18A	0.6488	0.2520	0.1518	0.085*	
C19	0.5788 (5)	0.1938 (4)	0.2909 (5)	0.124 (2)	
H19A	0.5052	0.2252	0.2818	0.186*	
H19B	0.5880	0.1937	0.3545	0.186*	
H19C	0.5895	0.1260	0.2852	0.186*	
C20	0.7770 (4)	0.2024 (4)	0.2251 (5)	0.122 (2)	
H20A	0.8281	0.2407	0.1749	0.183*	
H20B	0.7879	0.1350	0.2180	0.183*	
H20C	0.7898	0.2011	0.2879	0.183*	
C21	1.0901 (3)	0.2388 (5)	−0.1218 (4)	0.1077 (18)	
H21A	1.1288	0.1967	−0.1643	0.162*	
H21B	1.0837	0.2002	−0.0552	0.162*	
H21C	1.1306	0.2935	−0.1316	0.162*	
C22	0.3224 (6)	1.0398 (4)	−0.1290 (6)	0.149 (3)	
H22A	0.2484	1.0225	−0.1067	0.224*	
H22B	0.3392	1.0574	−0.1991	0.224*	
H22C	0.3276	1.0959	−0.1057	0.224*	
C32	0.1632 (3)	0.0901 (3)	0.5589 (3)	0.0721 (12)	
H32A	0.0931	0.0731	0.5649	0.086*	
C24	0.2062 (3)	−0.2782 (3)	0.5246 (3)	0.0597 (9)	
C34	0.2810 (3)	0.1747 (3)	0.6079 (3)	0.0624 (9)	
C35	0.3702 (3)	0.1407 (3)	0.5440 (3)	0.0639 (10)	
H35A	0.4406	0.1568	0.5386	0.077*	
C33	0.1773 (3)	0.1485 (4)	0.6153 (3)	0.0782 (13)	
H33A	0.1167	0.1706	0.6588	0.094*	
C29	0.1018 (3)	−0.2839 (3)	0.5090 (3)	0.0752 (11)	
H29A	0.0624	−0.2278	0.4735	0.090*	
C25	0.2597 (4)	−0.3628 (4)	0.5797 (4)	0.0933 (15)	
H25A	0.3296	−0.3608	0.5905	0.112*	
C27	0.1138 (6)	−0.4590 (4)	0.6033 (4)	0.1123 (19)	
C40	0.1482 (4)	0.2670 (3)	0.2820 (4)	0.0864 (13)	
H40A	0.0876	0.2683	0.3386	0.104*	
C28	0.0569 (5)	−0.3729 (4)	0.5465 (5)	0.1047 (17)	
H28A	−0.0118	−0.3765	0.5342	0.126*	
C43	0.3887 (4)	0.2620 (4)	0.6653 (4)	0.0995 (16)	
H43A	0.3788	0.3025	0.7107	0.149*	
H43B	0.4163	0.3000	0.6007	0.149*	
H43C	0.4410	0.2031	0.6830	0.149*	
C42	0.2333 (6)	0.3271 (5)	0.2858 (7)	0.152 (3)	
H42A	0.2601	0.2996	0.3459	0.229*	
H42B	0.2000	0.3956	0.2821	0.229*	
H42C	0.2943	0.3247	0.2318	0.229*	
C26	0.2137 (7)	−0.4490 (4)	0.6187 (4)	0.116 (2)	
H26A	0.2521	−0.5032	0.6574	0.139*	
C37	0.1099 (4)	−0.0422 (4)	0.2348 (4)	0.1015 (17)	
H37A	0.0467	−0.0566	0.2888	0.122*	
C38	0.1784 (9)	−0.1388 (5)	0.2208 (6)	0.193 (4)	
H38A	0.2058	−0.1746	0.2793	0.289*	
H38B	0.2399	−0.1251	0.1675	0.289*	
H38C	0.1334	−0.1789	0.2066	0.289*	
C41	0.1017 (7)	0.3054 (6)	0.1941 (6)	0.166 (3)	
H41A	0.0466	0.2654	0.1962	0.248*	
H41B	0.1598	0.3025	0.1382	0.248*	
H41C	0.0678	0.3738	0.1899	0.248*	
C44	0.0581 (8)	−0.5530 (5)	0.6473 (6)	0.179 (4)	
H44A	0.1063	−0.6044	0.6835	0.269*	
H44B	0.0434	−0.5760	0.5960	0.269*	
H44C	−0.0107	−0.5380	0.6901	0.269*	
C39	0.0687 (9)	0.0138 (8)	0.1473 (8)	0.202 (4)	
H39A	0.0215	0.0737	0.1588	0.304*	
H39B	0.0270	−0.0267	0.1298	0.304*	
H39C	0.1303	0.0315	0.0952	0.304*	
C30	0.2356 (2)	−0.0021 (3)	0.4265 (3)	0.0536 (8)	
H30A	0.3088	−0.0178	0.3871	0.064*	
C31	0.2520 (3)	0.0561 (3)	0.4933 (2)	0.0523 (8)	
C23	0.2607 (3)	−0.1856 (3)	0.4811 (3)	0.0603 (9)	
C36	0.3538 (3)	0.0825 (3)	0.4882 (3)	0.0624 (10)	
H36A	0.4146	0.0600	0.4451	0.075*	

### Atomic displacement parameters (Å^2^)

**Table:**

	*U*^11^	*U*^22^	*U*^33^	*U*^12^	*U*^13^	*U*^23^
P1	0.0486 (5)	0.0586 (6)	0.0503 (5)	0.0021 (4)	−0.0198 (4)	−0.0141 (4)
N1	0.0504 (15)	0.0487 (16)	0.0650 (17)	−0.0026 (12)	−0.0260 (13)	−0.0193 (14)
C1	0.061 (2)	0.048 (2)	0.087 (3)	−0.0059 (17)	−0.026 (2)	−0.0163 (19)
O1	0.105 (2)	0.080 (2)	0.213 (4)	−0.0052 (18)	−0.099 (3)	−0.049 (2)
O2	0.0510 (12)	0.0623 (15)	0.0600 (14)	−0.0056 (11)	−0.0191 (11)	−0.0131 (12)
C2	0.065 (2)	0.0392 (19)	0.072 (2)	0.0051 (16)	−0.0234 (18)	−0.0160 (18)
P2	0.0508 (5)	0.0632 (6)	0.0555 (6)	−0.0126 (4)	−0.0074 (4)	−0.0133 (5)
N2	0.0352 (13)	0.0492 (16)	0.0709 (18)	0.0023 (12)	−0.0046 (12)	−0.0112 (14)
O3	0.0563 (14)	0.0921 (19)	0.0739 (17)	−0.0027 (13)	−0.0116 (12)	−0.0423 (15)
C3	0.090 (3)	0.058 (3)	0.132 (4)	−0.014 (2)	−0.017 (3)	−0.003 (3)
O4	0.0776 (16)	0.0635 (17)	0.0567 (15)	0.0014 (13)	−0.0327 (12)	−0.0068 (13)
C4	0.149 (5)	0.052 (3)	0.131 (5)	−0.023 (3)	−0.027 (4)	0.006 (3)
O5	0.0500 (14)	0.098 (2)	0.0844 (19)	0.0083 (13)	−0.0195 (13)	−0.0336 (17)
C5	0.129 (4)	0.064 (3)	0.090 (3)	0.017 (3)	−0.038 (3)	−0.018 (3)
C6	0.075 (3)	0.079 (3)	0.091 (3)	0.017 (2)	−0.032 (2)	−0.024 (3)
O6	0.0475 (15)	0.089 (2)	0.137 (3)	0.0119 (14)	−0.0110 (16)	−0.044 (2)
C7	0.064 (2)	0.058 (2)	0.069 (2)	0.0051 (18)	−0.0203 (18)	−0.0152 (19)
O7	0.0480 (13)	0.0694 (16)	0.0711 (16)	−0.0115 (11)	−0.0142 (11)	−0.0093 (13)
C8	0.0491 (18)	0.0483 (19)	0.064 (2)	−0.0021 (15)	−0.0311 (16)	−0.0108 (17)
O8	0.0774 (18)	0.127 (3)	0.086 (2)	−0.0213 (18)	−0.0065 (16)	−0.055 (2)
C9	0.0367 (15)	0.0476 (18)	0.0548 (19)	−0.0049 (13)	−0.0165 (13)	−0.0079 (15)
O9	0.0662 (16)	0.0706 (19)	0.0833 (19)	−0.0164 (14)	−0.0048 (14)	0.0021 (15)
C10	0.0402 (16)	0.067 (2)	0.065 (2)	0.0009 (15)	−0.0262 (16)	−0.0196 (19)
O10	0.0582 (15)	0.119 (2)	0.092 (2)	−0.0230 (15)	−0.0044 (14)	−0.055 (2)
C11	0.0476 (18)	0.088 (3)	0.071 (2)	−0.0031 (18)	−0.0211 (17)	−0.033 (2)
C12	0.0394 (17)	0.064 (2)	0.059 (2)	−0.0008 (16)	−0.0118 (15)	−0.0132 (18)
C13	0.0423 (17)	0.080 (3)	0.067 (2)	0.0062 (17)	−0.0240 (16)	−0.017 (2)
C14	0.0512 (19)	0.078 (3)	0.066 (2)	−0.0009 (17)	−0.0291 (17)	−0.024 (2)
C15	0.064 (2)	0.102 (3)	0.069 (3)	−0.009 (2)	−0.011 (2)	−0.037 (3)
C16	0.110 (4)	0.120 (4)	0.137 (5)	0.021 (3)	−0.032 (4)	−0.074 (4)
C17	0.146 (5)	0.149 (6)	0.098 (4)	−0.017 (5)	0.008 (4)	−0.016 (4)
C18	0.073 (2)	0.074 (3)	0.062 (2)	0.007 (2)	−0.0171 (19)	−0.020 (2)
C19	0.126 (4)	0.093 (4)	0.142 (5)	−0.032 (4)	−0.003 (4)	−0.021 (4)
C20	0.097 (4)	0.094 (4)	0.144 (5)	0.032 (3)	−0.024 (3)	−0.014 (4)
C21	0.062 (2)	0.161 (5)	0.092 (3)	0.049 (3)	−0.027 (2)	−0.053 (4)
C22	0.194 (7)	0.078 (4)	0.163 (6)	0.058 (4)	−0.087 (5)	−0.020 (4)
C32	0.0389 (18)	0.119 (4)	0.069 (2)	−0.024 (2)	0.0019 (17)	−0.042 (3)
C24	0.071 (2)	0.053 (2)	0.051 (2)	0.0067 (18)	−0.0120 (17)	−0.0166 (18)
C34	0.057 (2)	0.073 (2)	0.060 (2)	−0.0138 (18)	−0.0154 (17)	−0.014 (2)
C35	0.0405 (17)	0.080 (3)	0.075 (2)	−0.0179 (17)	−0.0078 (17)	−0.023 (2)
C33	0.0454 (19)	0.125 (4)	0.077 (3)	−0.015 (2)	0.0028 (18)	−0.055 (3)
C29	0.073 (3)	0.057 (2)	0.085 (3)	−0.009 (2)	−0.011 (2)	−0.004 (2)
C25	0.119 (4)	0.070 (3)	0.097 (3)	0.025 (3)	−0.056 (3)	−0.028 (3)
C27	0.161 (6)	0.068 (3)	0.096 (4)	−0.017 (4)	−0.010 (4)	−0.015 (3)
C40	0.077 (3)	0.071 (3)	0.106 (4)	−0.015 (2)	−0.021 (3)	−0.008 (3)
C28	0.100 (4)	0.080 (4)	0.128 (4)	−0.022 (3)	−0.005 (3)	−0.024 (3)
C43	0.092 (3)	0.134 (4)	0.100 (3)	−0.054 (3)	−0.016 (3)	−0.048 (3)
C42	0.135 (5)	0.109 (5)	0.229 (9)	−0.050 (4)	−0.067 (5)	−0.018 (5)
C26	0.201 (7)	0.059 (3)	0.086 (4)	0.007 (4)	−0.060 (4)	−0.006 (3)
C37	0.096 (3)	0.125 (4)	0.096 (4)	−0.043 (3)	0.006 (3)	−0.050 (3)
C38	0.321 (12)	0.109 (5)	0.152 (7)	−0.009 (6)	−0.014 (7)	−0.078 (5)
C41	0.182 (7)	0.158 (6)	0.152 (6)	−0.030 (5)	−0.102 (6)	0.032 (5)
C44	0.253 (9)	0.069 (4)	0.182 (8)	−0.060 (5)	0.012 (7)	0.000 (4)
C39	0.235 (10)	0.213 (10)	0.202 (9)	0.003 (8)	−0.149 (8)	−0.054 (8)
C30	0.0362 (15)	0.058 (2)	0.059 (2)	−0.0048 (14)	0.0034 (14)	−0.0143 (17)
C31	0.0452 (17)	0.056 (2)	0.0528 (19)	−0.0090 (15)	−0.0106 (15)	−0.0064 (16)
C23	0.049 (2)	0.068 (3)	0.063 (2)	0.0026 (18)	−0.0127 (17)	−0.022 (2)
C36	0.0383 (17)	0.077 (3)	0.071 (2)	−0.0113 (17)	−0.0067 (16)	−0.018 (2)

### Geometric parameters (Å, º)

**Table:**

P1—O2	1.470 (2)	C19—H19A	0.9600
P1—O4	1.560 (3)	C19—H19B	0.9600
P1—O3	1.582 (3)	C19—H19C	0.9600
P1—C8	1.820 (3)	C20—H20A	0.9600
N1—C1	1.343 (4)	C20—H20B	0.9600
N1—C8	1.466 (4)	C20—H20C	0.9600
N1—H1A	0.8600	C21—H21A	0.9600
C1—O1	1.225 (4)	C21—H21B	0.9600
C1—C2	1.483 (5)	C21—H21C	0.9600
C2—C3	1.372 (6)	C22—H22A	0.9600
C2—C7	1.385 (5)	C22—H22B	0.9600
P2—O7	1.460 (2)	C22—H22C	0.9600
P2—O8	1.570 (3)	C32—C33	1.374 (5)
P2—O9	1.561 (3)	C32—C31	1.387 (5)
P2—C30	1.820 (4)	C32—H32A	0.9300
N2—C23	1.347 (4)	C24—C25	1.376 (6)
N2—C30	1.457 (4)	C24—C29	1.396 (5)
N2—H2A	0.8600	C24—C23	1.494 (5)
O3—C15	1.444 (4)	C34—C33	1.387 (5)
C3—C4	1.398 (7)	C34—C35	1.375 (5)
C3—H3A	0.9300	C35—C36	1.377 (5)
O4—C18	1.444 (5)	C35—H35A	0.9300
C4—C5	1.381 (8)	C33—H33A	0.9300
C4—H4A	0.9300	C29—C28	1.384 (6)
O5—C12	1.364 (4)	C29—H29A	0.9300
O5—C21	1.402 (5)	C25—C26	1.357 (7)
C5—C6	1.367 (7)	C25—H25A	0.9300
C5—C22	1.498 (7)	C27—C26	1.359 (8)
C6—C7	1.387 (5)	C27—C28	1.420 (8)
C6—H6A	0.9300	C27—C44	1.518 (8)
O6—C23	1.225 (4)	C40—C41	1.450 (8)
C7—H7A	0.9300	C40—C42	1.502 (7)
C8—C9	1.513 (5)	C40—H40A	0.9800
C8—H8A	0.9800	C28—H28A	0.9300
O8—C37	1.392 (5)	C43—H43A	0.9600
C9—C10	1.380 (4)	C43—H43B	0.9600
C9—C14	1.373 (4)	C43—H43C	0.9600
O9—C40	1.417 (5)	C42—H42A	0.9600
C10—C11	1.374 (5)	C42—H42B	0.9600
C10—H10A	0.9300	C42—H42C	0.9600
O10—C34	1.372 (4)	C26—H26A	0.9300
O10—C43	1.421 (5)	C37—C39	1.444 (10)
C11—C12	1.388 (5)	C37—C38	1.493 (9)
C11—H11A	0.9300	C37—H37A	0.9800
C12—C13	1.384 (5)	C38—H38A	0.9600
C13—C14	1.397 (5)	C38—H38B	0.9600
C13—H13A	0.9300	C38—H38C	0.9600
C14—H14A	0.9300	C41—H41A	0.9600
C15—C17	1.471 (8)	C41—H41B	0.9600
C15—C16	1.492 (7)	C41—H41C	0.9600
C15—H15A	0.9800	C44—H44A	0.9600
C16—H16A	0.9600	C44—H44B	0.9600
C16—H16B	0.9600	C44—H44C	0.9600
C16—H16C	0.9600	C39—H39A	0.9600
C17—H17A	0.9600	C39—H39B	0.9600
C17—H17B	0.9600	C39—H39C	0.9600
C17—H17C	0.9600	C30—C31	1.506 (5)
C18—C19	1.463 (7)	C30—H30A	0.9800
C18—C20	1.489 (6)	C31—C36	1.374 (4)
C18—H18A	0.9800	C36—H36A	0.9300
			
O2—P1—O4	115.52 (14)	O5—C21—H21A	109.5
O2—P1—O3	116.31 (13)	O5—C21—H21B	109.5
O4—P1—O3	103.65 (14)	H21A—C21—H21B	109.5
O2—P1—C8	112.92 (14)	O5—C21—H21C	109.5
O4—P1—C8	106.08 (14)	H21A—C21—H21C	109.5
O3—P1—C8	100.76 (15)	H21B—C21—H21C	109.5
C1—N1—C8	121.5 (3)	C5—C22—H22A	109.5
C1—N1—H1A	119.3	C5—C22—H22B	109.5
C8—N1—H1A	119.3	H22A—C22—H22B	109.5
O1—C1—N1	122.2 (4)	C5—C22—H22C	109.5
O1—C1—C2	121.5 (3)	H22A—C22—H22C	109.5
N1—C1—C2	116.3 (3)	H22B—C22—H22C	109.5
C3—C2—C7	118.7 (4)	C33—C32—C31	120.9 (3)
C3—C2—C1	120.3 (4)	C33—C32—H32A	119.6
C7—C2—C1	120.9 (3)	C31—C32—H32A	119.6
O7—P2—O8	116.08 (15)	C25—C24—C29	117.6 (4)
O7—P2—O9	117.14 (15)	C25—C24—C23	120.5 (4)
O8—P2—O9	100.74 (17)	C29—C24—C23	121.9 (3)
O7—P2—C30	113.59 (15)	O10—C34—C33	115.5 (3)
O8—P2—C30	103.84 (17)	O10—C34—C35	125.3 (3)
O9—P2—C30	103.56 (15)	C33—C34—C35	119.2 (3)
C23—N2—C30	121.6 (3)	C36—C35—C34	119.0 (3)
C23—N2—H2A	119.2	C36—C35—H35A	120.5
C30—N2—H2A	119.2	C34—C35—H35A	120.5
C15—O3—P1	124.0 (2)	C32—C33—C34	120.8 (3)
C2—C3—C4	120.8 (5)	C32—C33—H33A	119.6
C2—C3—H3A	119.6	C34—C33—H33A	119.6
C4—C3—H3A	119.6	C28—C29—C24	120.0 (4)
C18—O4—P1	124.5 (2)	C28—C29—H29A	120.0
C5—C4—C3	120.8 (5)	C24—C29—H29A	120.0
C5—C4—H4A	119.6	C24—C25—C26	122.1 (5)
C3—C4—H4A	119.6	C24—C25—H25A	118.9
C12—O5—C21	116.9 (3)	C26—C25—H25A	118.9
C6—C5—C4	117.5 (4)	C26—C27—C28	116.6 (5)
C6—C5—C22	121.0 (6)	C26—C27—C44	124.3 (7)
C4—C5—C22	121.5 (6)	C28—C27—C44	119.1 (7)
C7—C6—C5	122.6 (4)	O9—C40—C41	110.9 (5)
C7—C6—H6A	118.7	O9—C40—C42	107.2 (4)
C5—C6—H6A	118.7	C41—C40—C42	112.9 (5)
C6—C7—C2	119.6 (4)	O9—C40—H40A	108.6
C6—C7—H7A	120.2	C41—C40—H40A	108.6
C2—C7—H7A	120.2	C42—C40—H40A	108.6
N1—C8—C9	115.1 (3)	C29—C28—C27	121.2 (5)
N1—C8—P1	107.4 (2)	C29—C28—H28A	119.4
C9—C8—P1	111.9 (2)	C27—C28—H28A	119.4
N1—C8—H8A	107.3	O10—C43—H43A	109.5
C9—C8—H8A	107.3	O10—C43—H43B	109.5
P1—C8—H8A	107.3	H43A—C43—H43B	109.5
C37—O8—P2	128.1 (3)	O10—C43—H43C	109.5
C10—C9—C14	118.2 (3)	H43A—C43—H43C	109.5
C10—C9—C8	121.1 (3)	H43B—C43—H43C	109.5
C14—C9—C8	120.7 (3)	C40—C42—H42A	109.5
C40—O9—P2	126.3 (3)	C40—C42—H42B	109.5
C9—C10—C11	120.3 (3)	H42A—C42—H42B	109.5
C9—C10—H10A	119.9	C40—C42—H42C	109.5
C11—C10—H10A	119.9	H42A—C42—H42C	109.5
C34—O10—C43	118.7 (3)	H42B—C42—H42C	109.5
C10—C11—C12	121.5 (3)	C27—C26—C25	122.4 (5)
C10—C11—H11A	119.2	C27—C26—H26A	118.8
C12—C11—H11A	119.2	C25—C26—H26A	118.8
O5—C12—C11	116.3 (3)	O8—C37—C39	109.9 (6)
O5—C12—C13	124.7 (3)	O8—C37—C38	108.8 (6)
C11—C12—C13	119.0 (3)	C39—C37—C38	110.6 (6)
C14—C13—C12	118.4 (3)	O8—C37—H37A	109.2
C14—C13—H13A	120.8	C39—C37—H37A	109.2
C12—C13—H13A	120.8	C38—C37—H37A	109.2
C13—C14—C9	122.5 (3)	C37—C38—H38A	109.5
C13—C14—H14A	118.7	C37—C38—H38B	109.5
C9—C14—H14A	118.7	H38A—C38—H38B	109.5
O3—C15—C17	110.2 (4)	C37—C38—H38C	109.5
O3—C15—C16	106.0 (4)	H38A—C38—H38C	109.5
C17—C15—C16	113.1 (5)	H38B—C38—H38C	109.5
O3—C15—H15A	109.1	C40—C41—H41A	109.5
C17—C15—H15A	109.1	C40—C41—H41B	109.5
C16—C15—H15A	109.1	H41A—C41—H41B	109.5
C15—C16—H16A	109.5	C40—C41—H41C	109.5
C15—C16—H16B	109.5	H41A—C41—H41C	109.5
H16A—C16—H16B	109.5	H41B—C41—H41C	109.5
C15—C16—H16C	109.5	C27—C44—H44A	109.5
H16A—C16—H16C	109.5	C27—C44—H44B	109.5
H16B—C16—H16C	109.5	H44A—C44—H44B	109.5
C15—C17—H17A	109.5	C27—C44—H44C	109.5
C15—C17—H17B	109.5	H44A—C44—H44C	109.5
H17A—C17—H17B	109.5	H44B—C44—H44C	109.5
C15—C17—H17C	109.5	C37—C39—H39A	109.5
H17A—C17—H17C	109.5	C37—C39—H39B	109.5
H17B—C17—H17C	109.5	H39A—C39—H39B	109.5
O4—C18—C19	109.4 (4)	C37—C39—H39C	109.5
O4—C18—C20	108.3 (4)	H39A—C39—H39C	109.5
C19—C18—C20	112.0 (5)	H39B—C39—H39C	109.5
O4—C18—H18A	109.1	N2—C30—C31	115.4 (3)
C19—C18—H18A	109.1	N2—C30—P2	107.5 (2)
C20—C18—H18A	109.1	C31—C30—P2	113.2 (2)
C18—C19—H19A	109.5	N2—C30—H30A	106.8
C18—C19—H19B	109.5	C31—C30—H30A	106.8
H19A—C19—H19B	109.5	P2—C30—H30A	106.8
C18—C19—H19C	109.5	C36—C31—C32	117.1 (3)
H19A—C19—H19C	109.5	C36—C31—C30	121.7 (3)
H19B—C19—H19C	109.5	C32—C31—C30	121.1 (3)
C18—C20—H20A	109.5	O6—C23—N2	122.3 (3)
C18—C20—H20B	109.5	O6—C23—C24	121.1 (3)
H20A—C20—H20B	109.5	N2—C23—C24	116.6 (3)
C18—C20—H20C	109.5	C31—C36—C35	123.1 (3)
H20A—C20—H20C	109.5	C31—C36—H36A	118.4
H20B—C20—H20C	109.5	C35—C36—H36A	118.4
			
C8—N1—C1—O1	7.1 (6)	C10—C9—C14—C13	−3.5 (6)
C8—N1—C1—C2	−171.3 (3)	C8—C9—C14—C13	175.1 (3)
O1—C1—C2—C3	39.0 (6)	P1—O3—C15—C17	95.6 (5)
N1—C1—C2—C3	−142.5 (4)	P1—O3—C15—C16	−141.7 (4)
O1—C1—C2—C7	−137.4 (4)	P1—O4—C18—C19	−96.1 (4)
N1—C1—C2—C7	41.1 (5)	P1—O4—C18—C20	141.6 (4)
O2—P1—O3—C15	28.2 (4)	C43—O10—C34—C33	179.2 (4)
O4—P1—O3—C15	−99.7 (3)	C43—O10—C34—C35	−0.1 (6)
C8—P1—O3—C15	150.7 (3)	O10—C34—C35—C36	−180.0 (4)
C7—C2—C3—C4	−0.1 (7)	C33—C34—C35—C36	0.7 (6)
C1—C2—C3—C4	−176.6 (5)	C31—C32—C33—C34	−0.1 (7)
O2—P1—O4—C18	17.7 (3)	O10—C34—C33—C32	−180.0 (4)
O3—P1—O4—C18	146.2 (3)	C35—C34—C33—C32	−0.6 (7)
C8—P1—O4—C18	−108.2 (3)	C25—C24—C29—C28	−1.5 (6)
C2—C3—C4—C5	1.5 (9)	C23—C24—C29—C28	176.6 (4)
C3—C4—C5—C6	−2.0 (9)	C29—C24—C25—C26	−0.5 (6)
C3—C4—C5—C22	179.0 (6)	C23—C24—C25—C26	−178.6 (4)
C4—C5—C6—C7	1.1 (7)	P2—O9—C40—C41	−90.9 (5)
C22—C5—C6—C7	−179.8 (5)	P2—O9—C40—C42	145.5 (4)
C5—C6—C7—C2	0.3 (6)	C24—C29—C28—C27	1.7 (7)
C3—C2—C7—C6	−0.8 (6)	C26—C27—C28—C29	0.0 (8)
C1—C2—C7—C6	175.7 (4)	C44—C27—C28—C29	176.7 (5)
C1—N1—C8—C9	−109.7 (4)	C28—C27—C26—C25	−2.1 (9)
C1—N1—C8—P1	124.9 (3)	C44—C27—C26—C25	−178.6 (6)
O2—P1—C8—N1	55.7 (2)	C24—C25—C26—C27	2.4 (8)
O4—P1—C8—N1	−176.9 (2)	P2—O8—C37—C39	105.7 (6)
O3—P1—C8—N1	−69.1 (2)	P2—O8—C37—C38	−133.0 (5)
O2—P1—C8—C9	−71.6 (2)	C23—N2—C30—C31	−102.7 (4)
O4—P1—C8—C9	55.8 (2)	C23—N2—C30—P2	130.1 (3)
O3—P1—C8—C9	163.6 (2)	O7—P2—C30—N2	55.0 (3)
O7—P2—O8—C37	−14.4 (5)	O8—P2—C30—N2	−72.0 (2)
O9—P2—O8—C37	−142.0 (4)	O9—P2—C30—N2	−176.9 (2)
C30—P2—O8—C37	111.0 (4)	O7—P2—C30—C31	−73.5 (3)
N1—C8—C9—C10	−50.3 (4)	O8—P2—C30—C31	159.5 (2)
P1—C8—C9—C10	72.7 (4)	O9—P2—C30—C31	54.6 (3)
N1—C8—C9—C14	131.3 (3)	C33—C32—C31—C36	0.6 (6)
P1—C8—C9—C14	−105.7 (3)	C33—C32—C31—C30	−175.8 (4)
O7—P2—O9—C40	−0.1 (4)	N2—C30—C31—C36	122.0 (4)
O8—P2—O9—C40	126.8 (4)	P2—C30—C31—C36	−113.7 (3)
C30—P2—O9—C40	−126.0 (4)	N2—C30—C31—C32	−61.7 (4)
C14—C9—C10—C11	2.9 (5)	P2—C30—C31—C32	62.6 (4)
C8—C9—C10—C11	−175.6 (3)	C30—N2—C23—O6	4.4 (6)
C9—C10—C11—C12	0.0 (6)	C30—N2—C23—C24	−174.6 (3)
C21—O5—C12—C11	−178.0 (4)	C25—C24—C23—O6	35.2 (5)
C21—O5—C12—C13	2.9 (6)	C29—C24—C23—O6	−142.8 (4)
C10—C11—C12—O5	178.4 (3)	C25—C24—C23—N2	−145.7 (4)
C10—C11—C12—C13	−2.5 (6)	C29—C24—C23—N2	36.3 (5)
O5—C12—C13—C14	−179.0 (3)	C32—C31—C36—C35	−0.5 (6)
C11—C12—C13—C14	2.0 (6)	C30—C31—C36—C35	175.9 (4)
C12—C13—C14—C9	1.0 (6)	C34—C35—C36—C31	−0.2 (6)

### Hydrogen-bond geometry (Å, º)

**Table:**

*D*—H···*A*	*D*—H	H···*A*	*D*···*A*	*D*—H···*A*
N1—H1*A*···O2^i^	0.86	2.20	3.021 (4)	159
N2—H2*A*···O7^ii^	0.86	2.20	3.013 (4)	157

Symmetry codes: (i) −*x*+1, −*y*+1, −*z*; (ii) −*x*, −*y*, −*z*+1.
